# Fear of graft rejection 1–5 years after lung transplantation—A nationwide cohort study

**DOI:** 10.1002/nop2.184

**Published:** 2018-07-16

**Authors:** Anna Forsberg, Madeleine Nilsson, Sofie Jakobsson, Annette Lennerling, Annika Kisch

**Affiliations:** ^1^ Institute of Health Sciences Lund University Lund Sweden; ^2^ Department of Thoracic Transplantation and Cardiology Skåne University Hospital Skåne Sweden; ^3^ Queen Silvia Children´s Hospital, Sahlgrenska University Hospital Gothenburg Sweden; ^4^ Institute of Health and Care Sciences University of Gothenburg Gothenburg Sweden; ^5^ The Department of Transplantation Sahlgrenska University Hospital Gothenburg Sweden; ^6^ The Department of Haematology Skåne University Hospital Skåne Sweden

**Keywords:** graft rejection, lung transplantation, psychological well‐being, self‐efficacy

## Abstract

**Aim:**

To explore the perceived threat of the risk of graft rejection and its relationship to psychological general well‐being and self‐efficacy 1–5 years after lung transplantation.

**Design:**

A nationwide, cross‐sectional cohort study as a part of the Self‐management after thoracic transplantation study.

**Methods:**

A total of 117 lung transplant recipients due for their yearly follow‐up one (*N* = 35), two (*N* = 28), three (*N* = 23), four (*N* = 20) and 5 years (*N* = 11) after lung transplantation were included. We used three instruments; the Perceived Threat of the Risk of Graft Rejection, the Psychological General Well‐being and Self‐efficacy in chronic illness.

**Results:**

The lung recipients reported an overall low perceived threat of the risk of graft rejection with no gender differences. Intrusive anxiety explained 24.7% of the variance in the PGWB‐sum (*p *≤ 0.001) and makes a statistically significant (*β *= −497; *p *≤ 0.001) unique contribution to the overall psychological general well‐being (95%CI 3.004—1.515).

## 1 INTRODUCTION

Perceived Threat of the Risk of Graft Rejection (PTRGR) is prominent in organ transplant recipients’ lives (Nilsson, Persson, & Forsberg, 2008). When asked about what they fear most, the commonest response is graft rejection. The threat of graft rejection is not just a potential threat but one with a fairly high risk of occurring. If it occurs, it could irreversibly harm the transplanted organ.

There are no published data on lung recipients’ PTRGR despite the fact that cellular rejection caused by T cell‐mediated perivascular or bronchiolar mononuclear inflammation affects over 50% of LuTRs within the first year (Mangi et al., 2011). Most LuTRs will experience at least one acute rejection episode during the first post‐transplant year (Martinu, Dong‐Feng, & Palmer, 2009). The risk of rejection is highest in the first few months post‐transplant and decreases over time. Acute rejection is a risk factor for bronchiolitis obliterans syndrome (BOS). Currently, emphasis is placed on the prevention, early diagnosis and complete eradication of acute rejection episodes. The goal is to assure optimal short‐ and long‐term outcomes (Carney, Hobson, & McCalmont, 2017). The PTRGR exists in the tension between the immunosuppressive therapy prescribed to prevent graft rejection, symptoms caused by the side‐effects of the medication and adherence issues, where LuTRs might be tempted to be non‐adherent due to too many negative side‐effects despite being aware of the increased risk of graft rejection (Kugler et al., 2007).

When LuTRs were interviewed after transplantation, it was found that they strived to live normally (Dabbs et al., 2004). Striving to live normally was the core process involving symptom experience and interpretation associated with rejection. The development of rejection marked the beginning of the vulnerability stage. When rejection occurred, the LuTRs expressed surprise and disappointment. For nearly two decades, a Swedish research group has focused on recipients’ experiences of the specific event of graft rejection within the context of solid organ transplantation. The results of these studies (Forsberg, Bäckman, & Möller, 2000; Nilsson, 2010; Nilsson et al., 2008; Nilsson, Forsberg, Bäckman, Lenerling, & Persson, 2011; Nilsson, Forsberg, Lennerling, & Persson, 2013) have led to the development of a theoretical framework (Forsberg, Lennerling, Fridh, Karlsson, & Nilsson, 2015).

Organ transplant recipients (OTRs) expect damage to happen if graft rejection occurs, i.e., reduced function of their transplanted organ. One year after transplantation, liver transplant recipients experienced the threat of graft rejection as alternating between being something of no specific significance to fear of death. These feelings involved being constantly aware of their bodies, having a continual sense of fear, experiencing an invisible threat and being failed or simply “let down” by their bodies (Dabbs et al., 2004). Most OTRs make strong efforts to protect themselves from graft rejection, as reported by Nilsson et al. (2008), and about 33% fear that it will actually occur (Nilsson, 2010). When investigating kidney, liver and heart or lung recipients regarding graft‐related threat (GRT), Nilsson et al. (2010) reported that the patients’ scores were widely spread, 33% of the patients perceived a low level of GRT, 40% were uncertain and 27% experienced a high level of GRT. However, there were few lung recipients in the study by Nilsson et al. and those that participated were added to the heart recipients. The majority of the OTRs (74%) reported low levels of intrusive anxiety (IA). A high level of lack of control (LOC) was experienced by 48% (Nilsson, 2010). A reasonable assumption is that this perceived threat is also relevant for lung recipients and involves various psychological reactions, such as efforts to cope with the perceived threat. Therefore, the aim of the present study was to explore the PTRGR and its relationship to psychological general well‐being (PGWB) and self‐efficacy 1–5 years after lung transplantation. The key research question was: what are the characteristics of the experienced threat of the risk of graft rejection and related psychological reactions among lung transplant recipients (LuTRs)?

### 1.1 Design

This multicentre, cross‐sectional, cohort study is a part of the Swedish national Self‐management after thoracic transplantation (SMATT) study.

## 2 METHODS

### 2.1 Study population and instruments

The inclusion criteria were being a lung recipient due for the annual follow‐up 1–5 years after lung transplantation at either of the two Thoracic transplant centres in Sweden, Swedish speaking, mentally lucid, not hospitalized and without on‐going treatment for acute rejection. The main reasons for not being included were poor health status, declining participation and language.

In total, 117 out of 204 eligible lung transplant recipients due for their annual follow‐up were included at 1 year (*N* = 35), 2 years (*N* = 28), 3 years (*N* = 23), 4 years (*N* = 20) and 5 years (*N* = 11) after transplantation (57% of the eligible patients nationwide). Altogether, 112 (95%) lung recipients completed the three measurement instruments. Indications for transplantation and medications among the 117 included LuTRs are presented in Table 1.

**Table 1:** Clinical characteristics (*N* = 117)

	*N* (%)
Sex	
Female/male	59/58
Indications for transplantation	
Chronic obstructive pulmonary disease (COPD)	29 (24.8)
Lung fibrosis	24 (20.5)
Cystic fibrosis	19 (16.2)
Lack of Alpha 1‐ antitrypsin	19 (16.2)
Other	12 (10.2)
Pulmonary arterial hypertension	7 (6)
Emphysema	4 (3.4)
Bronchiectasis	3 (2.6)
Type of graft	
Double lung	98 (84.5)
Single lung	18 (15.5)
Immunosuppressive medication^a^	
Cyclosporine	61 (52)
Tacrolimus	45 (38.5)
Mycophenelate mofetil (MMF)	79 (67.5)
Azathioprine (AZA)	12 (10)
Steroids	63 (54)
Rapamycine	34 (29)

a Each participant had two or more immunosuppressive drugs.

The perceived threat of the risk of graft rejection was explored by the PTGR‐instrument, which measures the phenomenon by 12 items on a 5‐point Likert‐type scale (Nilsson et al., 2011). The meaning of the first factor, GRT, is a perception that the primary disease will return, leaving one as ill as before the transplantation and facing re‐transplantation. Thus, this factor shows the extent of the risk of anticipated harm and implications for the future. The total GRT‐score varies from 3‐15, where a score >9 indicates a strong belief that graft rejection is a serious threat. The second factor, IA, means being constantly aware of the risk of graft rejection and thinking about it all the time. It also means experiencing great anxiety, which is elevated when taking immunosuppressive medication or undergoing a biopsy. Thus, this factor shows the extent of the OTRs’ stress response and level of anxiety. The total IA‐score varies from 6 to 30, where a score >18 indicates great intrusion. Finally, the third factor, LOC, involves perceptions that the threat of the risk of graft rejection is beyond one's control, revealing the degree of belief that one can control and protect oneself from the threat. The total LOC‐score varies from 3 to 15, where a score >9 indicates the perception of low control over one's ability to do anything to reduce the risk of graft rejection. Inter‐item correlation values range from 0.72 to 0.89 and a Cronbach's Alpha ranges from 0.81 to 0.91(Nilsson et al., 2011).

The Swedish version of the PGWB instrument was used to explore psychological well‐being and illness (Wiklund & Karlberg, 1991) where Cronbach's Alpha ranges from 0.61 to 0.88. It contains 22 items constituting six dimensions, i.e., anxiety, depressed mood, positive well‐being, self‐control, general health and vitality. The PGWB sum score is 132 and a normal sum score is considered to be between 100 and 105. A score below 100 indicates poorer psychological well‐being (Dimenas, Carlsson, Glise, Israelsson, & Wiklund, 1996; Dupuy, 1984).

Self‐efficacy was studied by the *Self‐Efficacy for managing chronic disease* instrument developed by Stanford Patient Education Centre. Self‐efficacy is measured by one homogenous factor made up of six statements with inter‐item correlation values 0.78–0.90 and a Cronbach's Alpha of 0.92 (Freund, Gensichen, Goetz, Szecsdenyi, & Mahler, 2013). The maximum score is 10 (range 0–10).

### 2.2 Statistical analysis and ethics

The SPSS Statistics 23 (SPSS Inc**.**, IBM Corporation, Armonk, NY, USA) was used for analysing data, which were mainly ordinal. Single‐scale ordered category data were summarized with median and percentiles (P_25_, P_75_). We tested three main hypotheses or questions:

1. There is no difference in PTGR between men and women or between those younger or older than 50 years (Mann–Whitney U).
2. There is no relationship between PTGR and PGWB or Self‐efficacy (Spearman's rho).
3. If we control for the possible effect of age and sex, is IA still able to predict a significant amount of variance in PGWB and self‐efficacy? (Hierarchical multiple regression).

When applicable, values of *p* < 0.05 (two‐tailed) were considered statistically significant. The analysis was performed stepwise as follows:

1. Explore proportions and describe the distribution of PTGR as well as consequences for PGWB
2. Explore possible differences between two unpaired groups, e.g. men and women.
3. Explore possible relationships.
4. Analyse possible explanatory factors.

In the whole group (*N* = 117), the male/female ratio was similar with 59 women and 58 men. All analysis regarding PTRGR is based on *N* = 112, 55 women and 57 men, as five responses on the PTGR‐instrument were missing. Age was dichotomized into two groups, younger and older than 50 years. Hierarchical multiple regression was used to assess the ability of IA to predict the PGWB‐sum and self‐efficacy levels after controlling for the influence age and sex.

Permission to carry out this study was granted by the Regional Ethical Review Board of southern Sweden (D‐nr 2014–124). All participants gave their written informed consent and the information they provided was kept confidential and stored by the researchers in accordance with the Swedish personal data act; PuL‐[1998:204] (Swedish Personal Data Act, 2015).

## 3 RESULTS

**Figure 1:**
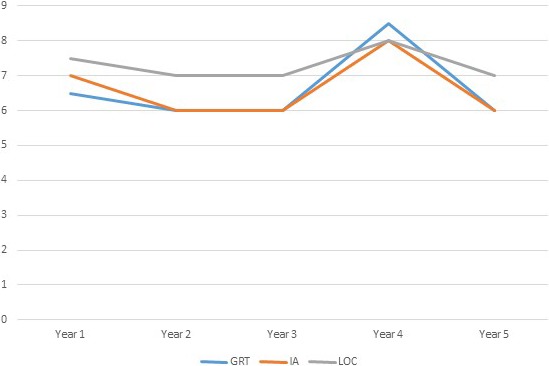
Median profile of the three dimensions of the perceived threat of the risk of graft rejection, i.e., graft‐related threat (GRT), intrusive anxiety (IA) and lack of control (LOC), each follow‐up year. The higher the median score, greater the fear or experienced intrusion

Of the LuTRs, 75% had scores that indicated a low perceived GRT with a median GRT 7 (P_25_ 4, P_75_ 9) and range 2–16. A total of 26 LuTRs (23%) scored above 9, indicating beliefs that graft rejection means returning to the pre‐transplant condition of severe illness. In terms of IA, the range was slightly wider (6–24). A median of 6 (P_25_ 6, P_75_ 9) showed that the perceived IA was low. Seven patients (6%) scored above 18, indicating problematic intrusion. Almost 75% of the participants reported that they perceived some sort of control over their ability to reduce the risk of graft rejection. The median LOC was 7 (P_25_ 4.5, P_75_ 9.75) with a range of 3–16. LuTRs at their 4‐year follow‐up reported higher GRT, IA and LOC than the rest of the recipients, where the perceived threat was generally experienced as low over the 5 years of follow‐up (Figure 1). There were no differences between men and women in GRT (*p *= 0.273), IA (*p *= 0.235) or LOC (*p *= 0.771). When dividing the whole group into two age groups <50 years (*N* = 31) and >50 years (*N* = 81), the older patients reported more IA (*p *= 0.007). However, there were no differences in GRT (*p* = 0. 356) or LOC (*p *= 0.431).

Relationships between the PTGR, PGWB and self‐efficacy over the 5 years are presented in Table 2. At the 1‐year follow‐up, there was a significant negative relationship between IA and all aspects of PGWB except vitality. The greater the experience of intrusion due to the risk of graft rejection, the lower the psychological well‐being. There was also a significant negative relationship between self‐efficacy and all three dimensions of the PTGR at the 1‐year follow‐up. Thus, the greater the experience of intrusion and GRT, the lower the perceived self‐efficacy. In addition when the LuTRs perceived a LOC over the risk of graft rejection (indicated by high LOC scores), they also reported low self‐efficacy. At the 2‐year follow‐up, no relationships were identified. At the 3‐year follow‐up, only one relationship was observed, namely a significant negative relationship between IA and self‐efficacy. However, a different pattern was revealed at the 4‐year and 5‐year follow‐ups. Intrusive anxiety was strongly related to almost all aspects of PGWB, thus the/greater the experience of intrusion, the stronger the anxiety and depression, leading to reduced well‐being, self‐control, vitality and general health. Intrusive anxiety explained 24.7% of the variance in the PGWB‐sum (*p* ≤ 0.001) and makes a statistically significant (*β *= −497; *p *≤ 0.001) unique contribution to the overall PGWB (95%CI 3.004–1.515). Using hierarchical multiple regression, we found that IA explains 40% of the variance in self‐efficacy after controlling for sex and age, thus making a statistically significant unique contribution to the overall perceived self‐efficacy (*β *= −407; *p *≤ 0.001). However, 4 and 5 years after lung transplantation, the relationship between PTGR and self‐efficacy was no longer significant.

**Table 2:** The relationship between the perceived threat of the risk of graft rejection (PTGR) and Psychological General Well‐being (PGWB) as well as Self‐efficacy in chronic illness 1–5 year after lung transplantation (*N* = 112)

PTGR each follow‐up	PGWB‐sum score	Anxiety	Depression	Psychological well‐being	Self‐control	General Health	Vitality	Self‐efficacy
1 year *N* = 32								
GRT	−0.337	−0.314	−0.162	−0.263	−0.293	−0.336	−0.245	−**0.473** **
IA	**−0.542** *	**−0.589** **	**−0.613** **	**−0.517** **	**−0.609** **	**−0.388** *	−260	**−0.571** **
LOC	−0.263	−0.176	‐0.343	**−0.353** *	−0.157	−0.224	−290	**−0.368** *
2 years *N* = 28								
GRT	0.056	0.193	0.029	−0.003	0.013	0.287	−0.074	0.293
IA	−0.290	−0.320	−0.268	−0.019	−0.319	−0.161	−0.287	−0.272
LOC	−0.161	−0.009	−0.200	−0.119	−0.276	−0.185	−0.074	−0.266
3 years *N* = 22								
GRT	−0.351	−0.385	−0.381	−0.275	−0.244	−0.363	−0.413	−0.482
IA	−0.245	−0.405	−0.257	−0.134	−0.054	−0.249	−0.241	−**0.570** **
LOC	0.015	−0.058	−0.344	−0.033	−0.154	0.010	0.006	0.022
4 years *N* = 19								
GRT	0.109	0.109	0.031	0.240	0.094	−0.106	0.167	0.203
IA	**−0.533** *	**−0.551** *	−0.243	−0.130	**−0.559** *	**−0.617** **	**−0.556** *	−0.259
LOC	−0.241	−0.310	0.046	0.009	−0.110	−0.288	−0.193	−0.099
5 years *N* = 11								
GRT	−0.401	−0.434	−0.229	−0.397	−0.051	−0.508	−0.353	−0.274
IA	−**0.820** **	−**0.780** **	−**0.872** **	−**801** **	−**0.644** *	−**0.776** **	−**790** **	−0.545
LOC	−0.587	−0.380	−0.400	−**0.641** *	−0.116	−**0.647** *	−0.490	−0.550

Note

The PTGR includes three dimensions, i.e., graft‐related threat (GRT), intrusive anxiety (IA) and lack of control (LOC). Correlation values that were significant are marked in bold.

* *p* < 0.005.

** *p* ≤ 0.001.

## 4 DISCUSSION

The main findings in this study were:

1. The PTGR expressed as GRT, IA and LOC was low 1–5 years after lung transplantation.
2. There were no gender differences in PTGR.
3. Lung recipients older than 50 years reported a higher level of IA than the younger recipients.
4. There was a significant relationship between IA and almost all dimensions of PGWB 1, 4 and 5** **years after lung transplantation.
5. Although the prevalence of problematic IA was low, it explains close to 25% of the variance in PGWB.

This is the first study that reports in detail how lung transplant recipients perceive the threat of the risk of graft rejection. To our satisfaction, the fear of graft rejection was low. This is in line with a previous study on other solid organ recipients (Nilsson et al., 2011) where 74% had low scores on IA. It suggests that LuTRs are capable of mastering the threat of graft rejection despite the high risk of occurrence (Mangi et al., 2011; Martinu et al., 2009). As illustrated in Table 2, there was a moderate relationship between IA and various aspects of PGWB at the 1‐year follow‐up, which is reasonable due to the high risk of graft rejection the first post‐transplant year. After 5 years, there was also a very strong relationship between IA and the psychological markers. This pattern might be related to the occurrence of BOS, as approximately 48% develop BOS during the first 5 years after LuTx and 76% after 10 years (Carney et al., 2017), leading to an increased mortality risk. Each acute rejection can lead to BOS, which precedes chronic lung allograft dysfunction (CLAD), end‐stage graft failure leading to either the need for re‐transplantation in selected cases or palliative care (Yusen et al., 2014).

We find it reasonable that there are no gender differences, as there is no evidence in the literature that women are more afraid of graft rejection then men. The fear experienced is more likely to be related to personality traits and coping strategies than sex. The fact that the younger patients perceived lower IA might be because they are occupied with various activities of everyday life, leaving little time for pondering on graft rejection.

The relationship between PGWB and the perceived threat of graft rejection was obvious. The explanation for this relationship might stem from the actual phenomenon of fear and threat implied in the concept of PTRGR as described in the introduction. Risk can be defined as exposure to the likelihood of a negative event and being an “at‐risk person”, i.e., being a lung transplant recipient means being unintentionally at risk of graft rejection (O´Byrne, 2008). Threat implies an indication of impending danger or harm; something that is regarded as a possible danger; a menace. The threat remains when harm has not happened but is expected (Carpenter, 2005). All lung recipients are educated about graft rejection and thus constantly aware that harm is to be expected sooner or later. Lazarus and Folkman (1984) also defined threat as being “a threatening encounter that makes one feel uneasy (anxious), which is connected with a strong effort to protect oneself from anticipated danger” (p. 18). The only way a LuTR can protect her/himself from graft rejection is by taking the immunosuppressive medication as prescribed. Furthermore, perceived threat is a threat based on a perception of some anticipated harm (Lazarus, 1991). The harm can take various forms such a perceived loss, interference with needs or goals, and perceived LOC. It is the individual's perception of the cue or event that is meaningful, not the kind or quality of the perceived anticipated harm. Thus, it is fairly reasonable that IA explains almost 25% of the PGWB after lung transplantation, despite the fact that the median IA was 6 and only seven patients (6%) scored above 18, indicating problematic IA. The fear of graft rejection might be viewed as a sort of uncertainty in illness that causes psychological distress as described among heart recipients (Almgren, Lennerling, Lundmark, & Forsberg, 2017). As the risk of graft rejection is inherent in being a lung recipient, support is needed to master the constant uncertainty in illness and develop self‐management strategies aimed at adherence to the immunosuppressive regimen. It is important to prevent the scenario described by Kugler et al. (2007) where organ recipients might be tempted to become non‐adherent due to the side‐effects of the medication.

An interesting but less pronounced finding was the relationship between the PTGR, especially IA, and self‐efficacy. Self‐efficacy, which means the perceived capability of the lung recipients to perform a specific action required to achieve a concrete goal, is lower when the PTGR is high. The experience of high IA might reduce the capability of performing actions. As self‐efficacy is an important part of self‐management, it is important to identify those recipients with an IA score >18 for further self‐management support. For this purpose, the theoretical framework suggested by Forsberg et al. (2015) might be highly useful in clinical practice, where an expert nurse practitioner or a transplant nurse could perform the assessment and provide the necessary support.

### 4.1 Methodological considerations

The limitations of this study are due to the cross‐sectional design. All the data were self‐reported, thus representing the inside perspective of the patients’ experiences. As a consequence, it allows different interpretations of the items. The recruitment of patients during the study period was probably affected by the different staffing conditions at the outpatient lung transplant clinic in the two thoracic transplant centres in Sweden. The slightly different follow‐up approach to the care of these patients in the pre, peri or/and postoperative setting contributes to the heterogeneity of the study population. Although this heterogeneity might be considered a weakness, it could also be viewed as a strength as it may more closely represent a cross‐section of the patients who undergo lung transplantation in Sweden.

The advantage of using the PTGR is that it is a transplant‐specific instrument with good psychometric properties. The PTGR is based on extensive research, resulting in a clear description of measurement aims, target population, the theoretical framework, item selection, item reduction and the workload required from respondents to complete the questionnaire (Nilsson et al., 2011). A limitation is that reliability is not yet fully tested regarding stability and sensitivity to change. The other two instruments used are well established with good psychometric properties. Together, the three instruments provide a detailed picture of the impact of fear of graft rejection in the lives of lung recipients up to 5 year after transplantation.

### 4.2 Conclusion and clinical implications

The fear of graft rejection after lung transplantation is a relatively insignificant problem with no gender differences. When it occurs it is mainly in the form of IA, which is strongly related to the patient's overall psychological well‐being. IA might hamper self‐efficacy at 1 and 3 years after lung transplantation. A recent systematic review on research priority setting in organ transplantation (Tong et al., 2017) reveals that stakeholders i.e., patients, caregivers, living kidney donors and health professionals, address graft‐related complications as an important research area. This involves acute rejection, graft function and chronic graft rejection. Thus, this research is considered important by those concerned.

The PTGR‐instrument could serve as a patient‐reported experience measure (PREM) (Black, 2013) and is a clinically useful tool with 12‐items that easily detect a possibly harmful relationship to the risk of graft rejection. PREMs focus on aspects of the humanity of care, such as being treated with dignity or not kept waiting (Black, 2013). Apart from the PTGR instrument, there are no PREMs currently used for measuring patients’ experiences of graft rejection. One reason might be a lack of inductively developed measures representing the inside perspective of the patient. However, there are several patient‐reported outcome measures (PROMs) available, e.g. measurement of pain, nausea, fatigue and distress. PROMs seek to ascertain patients’ views of their symptoms, functional status and health‐related quality of life (HRQoL). There are two types of PROM, disease‐specific and generic, of which there are thousands the former, but not within transplantation care (Garret, Schmidt, Mackintosh, & Fitzpatrick, 2002). In a short‐term perspective, PROMs provide feedback on immediate individual care, while PREMs enable feedback on the integration of care, thus allowing patients to drive improvement in services. There is an established framework that might support clinicians when assessing and intervening in relation to graft rejection, where transplant nurses and expert nurse practitioners can serve as the first‐line professionals. The main support for recipients with high IA and low psychological well‐being should be at the 1‐year follow‐up as well as 4–5 years after transplantation and probably at an even later stage.

## CONFLICT OF INTEREST

We have no conflict of interests.

## AUTHOR CONTRIBUTION

AF: Designed the study, collected and analysed data and drafted the manuscript. MN: Developed the PTGR‐instrument, prepared the manuscript and contributed important aspects of the fear of graft rejection. SJ: Prepared the manuscript and contributed important aspects of the fear of graft rejection. AL: Designed the study, collected data and prepared the manuscript. AK: Designed the study, prepared the manuscript and contributed important aspects of the fear of graft rejection.

All authors have agreed on the final version and meet at least one of the following criteria [recommended by the ICMJE (https://www.icmje.org/recommendations/ )]:

- substantial contributions to conception and design, acquisition of data or analysis and interpretation of data;
- drafting the article or revising it critically for important intellectual content.
